# Experimental demonstration of an arbitrary shape dc electric concentrator

**DOI:** 10.1038/s41598-020-73561-8

**Published:** 2020-10-07

**Authors:** Hooman Barati Sedeh, Mohammad Hosein Fakheri, Ali Abdolali, Fei Sun

**Affiliations:** 1 Applied Electromagnetic Laboratory, School of Electrical Engineering, Iran University of Science and Technology, Tehran, 1684613114 Iran; 2 College of Physics and Optoelectronics, Taiyuan University of Technology, Taiyuan, 030024 China

**Keywords:** Transformation optics, Electrical and electronic engineering, Metamaterials

## Abstract

Coordinate transformation (CT) theory has shown great potentials in manipulating both time-varying and static fields for different physics ranging from electromagnetism and acoustics to electrostatic and thermal science. Nevertheless, as inhomogeneous and anisotropic materials are required to be realized for the implementation of CT-based devices, the applicability of this method is restricted due to difficulties in the fabrication process. In this paper, based on transformation electrostatic (TE) methodology, the design principle of an arbitrary shape dc electric concentrator is established which yields the enhancement of static electric fields in a predefined region with only one homogeneous conductivity, named as dc null medium (DNM). It is shown that one constant DNM is sufficient for localizing steady electric current in any arbitrary shape region, which in turn obviates the tedious mathematical calculations that conventional methods suffer from. In other words, the same DNM can be used for different concentrators regardless of their cross-section geometries, which makes the presented approach suitable for scenarios where reconfigurability is of utmost importance. Several numerical simulations are performed in order to demonstrate the capability of the proposed dc electric concentrator in localizing steady electric fields into the desired region. Moreover, by utilizing the analogy between electrically conducting materials and resistor networks, the attained DNM is realized with low-cost resistors and then exploited for fabricating a square shape dc electric concentrator on a printed circuit board (PCB). It is demonstrated that the measurement results agree well with the theoretical predictions and numerical simulations, which corroborate the effectiveness of the propounded method. The presented idea of this paper could find applications in scenarios where highly confined electric fields/currents are of critical importance such as electronic skin devices and electrical impedance tomography.

## Introduction

Controlling electromagnetic (EM) fields to render an object invisible has been a long-standing dream for many scientists over decades. The seminal work of Pendry et al, named as transformation optics (TO), which was based on the form invariant of Maxwell’s equations exhibit great potentials to manipulate EM wave propagation arbitrarily^1^ and yields the introduction of many interesting devices which were deemed impossible to be achieved such as EM cloaks^2,3^, beam steering devices^4,5^ and collimating lenses^6,7^. Although static fields play a significant role in several applications such as in electric impedance tomography (EIT)^8^, electrostatic spraying systems^9^, and photocopy machines, most of TO devices were designed and fabricated in time-varying EM fields. In 2007, Wood et al., exploited coordinate transformation in static potentials and cloaking for the static magnetic field was then propounded based on transformation electrostatic (TE) methodology^10,11^. Besides cloak, dc electric concentrators^12,13^, which are devices that can concentrate static fields in a given region while the external fields keep undisturbed has attracted a lot of attention due to its potential applications in scenarios where uniform enhanced current density is of critical importance including electronic skin (e-skin) devices^14^. Sensors play a crucial role in tactile sensing which is necessary to enable skin-like functionality^14^. In particular, these sensors can be used to evaluate human motion, and vital signs such as heart rate, respiration, and body temperature for health monitoring. However, for a sensor to function accurately, the electric current is of critical importance. That is, a local well enhanced electric current density plays a crucial role to avoid malfunctions in e-skin devices. This can be achieved by utilizing dc electric concentrators as they can localize steady electric field and current in the predefined region of interest. In addition to e-skin devices, the core idea of dc electric concentrators have potential applications in electrical impedance tomography (EIT), which are noninvasive type of medical imaging systems that are used to form a tomographic image of the human body^8^. As most of the EIT systems apply electric currents for their measurement, local enhanced current densities that do not distort the voltage equipotential lines of the background medium are of utmost importance. However, typical applied currents in EIT are relatively small and certainly below the threshold at which they would result in severe nerve stimulation. Therefore, the potential use of electric concentrators in EIT systems is for applying a well-enhanced current while the background external fields and voltage equipotential lines are not distorted. However, to date, all the proposed TE-based dc concentrators are limited to circular cross-section geometries, which in turn limit the applicability of the designed devices for more general cases where there is a prerequisite to focus static electric field in an arbitrary domain. Besides the geometry problem, the main drawback of TE-based concentrators is their inhomogeneous and anisotropic conductivities, which are of paramount difficulty for the fabrication process. In addition to the realization procedure, the obtained materials from TE methodology possess a high dependency on the applied transformation function. That is, if the mapping function is changed (due to the alternation in the virtual and physical domain geometries), the necessitating conductivities must be recalculated and redesigned. That is why an arbitrary shape dc electric concentrator has never been demonstrated experimentally in time-varying and static fields^12,15–23^.

Recently, novel approaches for achieving arbitrary shape time-varying fields concentrators have been proposed that are based on new materials which named as optic null medium (ONM) and acoustic null medium (ANM)^24,25^. However, to the authors’ best knowledge, no systematic works for extending the concept of ONM/ANM into static fields have been performed. Therefore, following our previous theoretical works, in this paper, we will outline a new design principle to achieve arbitrary shape dc electric concentrators based on null transformation methodology, which leads to a new material that is named as dc null medium (DNM). It will be shown that the proposed DNM is independent of the concentrator geometries. That is, if the device shape is changed, one can still use the same DNM without the demand for recalculating a new material. Several numerical simulations are carried out to verify the validity of the proposed theoretical investigations. To authenticate the concept, with the aid of analogy between electrically conducting materials and resistor networks, which was reported previously by Yang et al.^26^, we have realized the DNM with low-cost resistors and exploited it to implement a square shape dc electric concentrator. To the authors’ best knowledge, it is the first time that an arbitrary shape dc electric concentrator is being realized with feasible material. It is observed that the experimental results exhibit good agreement with the theoretical predictions and numerical simulation results, which indicates the generality and effectiveness of the proposed designing method. We emphasize that in this work, our main concern is to point out the novel capability of DNM in manipulating electrostatic fields as well as demonstrating the electric field concentration in an arbitrary region. However, with the expeditious growth in technology and material science, it is expected that the presented idea of this work finds applications in scenarios where compactness is of critical importance such as non-destructive sensors and energy harvest devices.

## Results

### Theory

**Figure 1:**
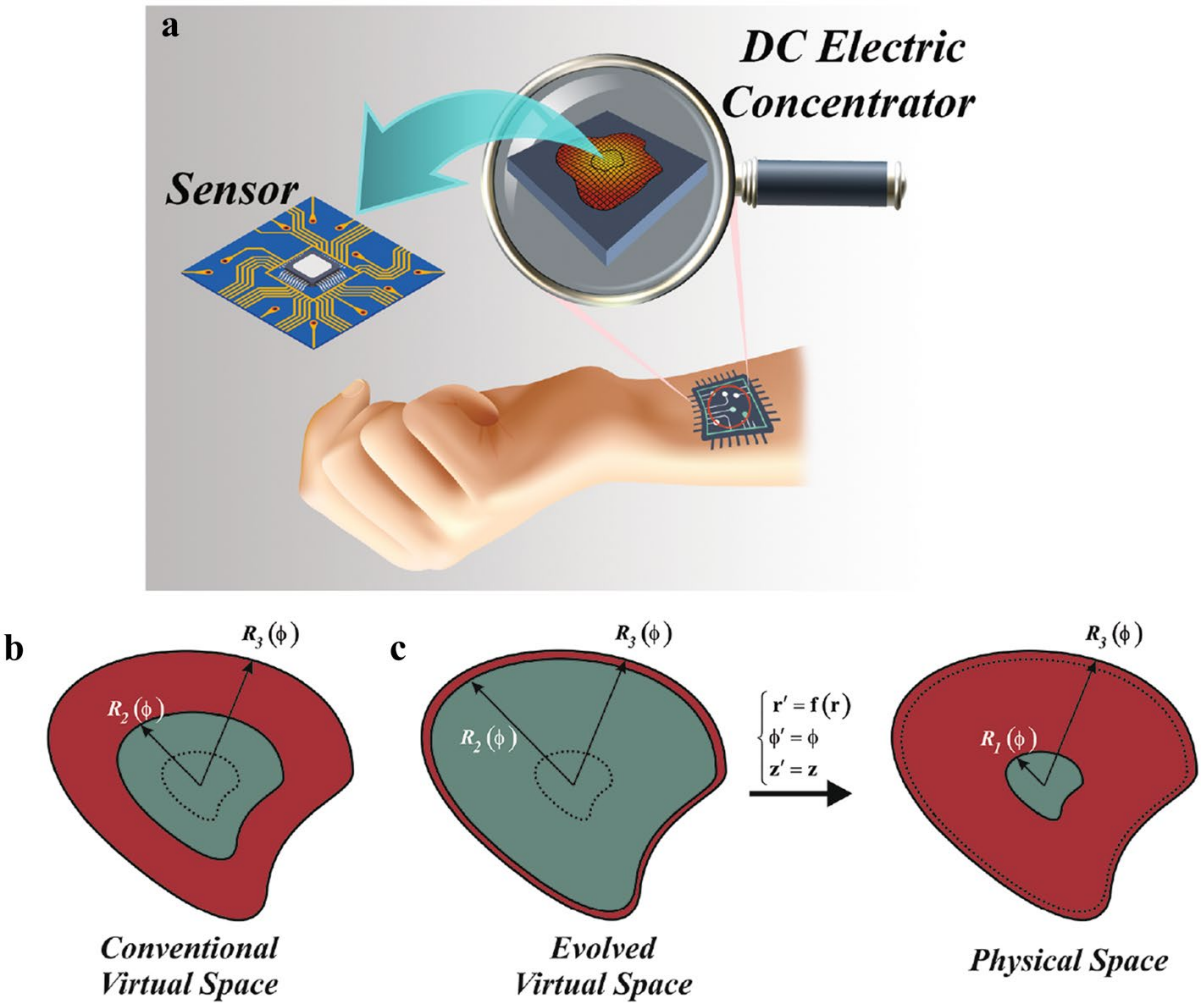
(**a**) The schematic of potential usage of a dc electric concentrator in e-skin applications. (**b**) The schematic of the conventional virtual space for achieving arbitrary shape concentrators with conformal boundaries. (**c**) The corresponding coordinate transformation when $R_2(\phi ) \rightarrow R_3(\phi )$.

The goal is to construct an electrostatic device that is competent in concentrating steady electric current density in an arbitrary region without influencing the potential distributions of the outside of the concentrator. Since in e-skin devices, the more the electric currents are enhanced, the more device sensitivity increases, which gives rise to better and accurate functionality, dc electric concentrator could have potential applications in e-skins as it is shown schematically in Fig. 1a.

To this aim, the region of $r \in [0,R_2(\phi )]$ must be compressed into $r^\prime \in [0,R_1(\phi ^\prime )]$ while $r \in [R_2(\phi ),R_3(\phi )]$ must be stretched into $r^\prime \in [R_1(\phi ^\prime ),R_3(\phi ^\prime )]$ as shown in Fig. 1b, which can be expressed mathematically as

1 $$\begin{aligned}&f_c(r,\phi )=\frac{R_1(\phi )}{R_2(\phi )}r, \nonumber \\&f_s(r,\phi )= \left( \frac{R_3(\phi )-R_1(\phi )}{R_3(\phi )-R_2(\phi )}\right) r + \left( \frac{R_1(\phi )-R_2(\phi )}{R_3(\phi )-R_2(\phi )}\right) R_3(\phi ) \end{aligned}$$

where subscripts of *c* and *s* represent the compressed (i.e., $r^\prime \in [0, R_1(\phi ))$) and stretched regions (i.e., $r^\prime \in [R_1 (\phi ), R_3 (\phi )]$), respectively. Therefore, the necessitating conductivities of the physical space can be obtained according to $\sigma ^\prime = (\det (\Lambda ))^{-1}\Lambda \sigma _0 \Lambda ^T$, where $\sigma _0$ is the background conductivity and $\Lambda$ is the Jacobian matrix, as $$\sigma ^\prime =\sigma _0 \begin{bmatrix} \sigma _{11} &{} \sigma _{12} &{} 0 \\ \sigma _{21} &{} \sigma _{22} &{} 0\\ 0 &{} 0&{} \sigma _{33} \end{bmatrix}$$, with

2 $$\begin{aligned}&\sigma _{11}= \left[ \frac{r}{f_i(r,\phi )} \left( \frac{\partial f_i(r,\phi )}{\partial r}\right) + \frac{\left( \partial f_i(r,\phi )/\partial \phi \right) ^2}{rr^\prime \left( \partial f_i(r,\phi )/\partial r\right) }\right] , \sigma _{12}=\sigma _{21}= \frac{1}{r} \times \left( \frac{\partial f_i(r,\phi )/\partial \phi }{\partial f_i(r,\phi )/\partial r}\right) , \sigma _{22}= \frac{f_i(r,\phi )}{r \left( \partial f_i(r,\phi )/\partial r\right) } \nonumber \\&\sigma _{33}= \frac{r}{f_i(r,\phi )\times \left( \partial f_i(r,\phi )/\partial r\right) } \end{aligned}$$

which $f_i(r,\phi )$ with $\textit{i}=\textit{c}$, *s* are the transformation functions defined in Eq. (1). It should be mentioned that since we are solving the problem for two-dimensional geometry, one can safely neglect the value of $\sigma _{33}$. Moreover, as the geometry of the region where the current density should be increased in (i.e., $r^\prime < R_1(\phi ^\prime )$ ) plays a crucial role, one can assume the contours are conformal as $R_1(\phi )=\tau _1 R(\phi )$, $R_2(\phi )=\tau _2 R(\phi )$ and $R_3(\phi )=\tau _3 R(\phi )$ where $\tau _i$ are constant coefficients and $R(\phi )$ is an arbitrary shape contour defined by the Fourier series. This can lead to a more simplified isotropic conductivity in the compressed region, as $f_c(r,\phi )$ would be independent of $\phi$ in this region (i.e., $f_c(r)=(\tau _1/\tau _2)r$). Nevertheless, in the stretched region, the off-diagonal components will remain due to the existence of $R(\phi )$ in the second term of $f_s(r,\phi )$. In other words, by applying such an assumption the conductivity tensor of the stretched region will be changed to

3 $$\begin{aligned}&\sigma _{11}= \frac{M_1(r^\prime ,\phi ^\prime )}{\delta }, \sigma _{22}= \delta M_2 (r^\prime , \phi ^\prime ), \sigma _{12}=\sigma _{21}= \frac{\tau _3 (\tau _1-\tau _2) \left( \partial R(\phi )/ \partial \phi \right) }{\delta r^\prime -\tau _3(\tau _1-\tau _2)R(\phi )} \end{aligned}$$

where $\delta = \tau _3-\tau _2$ and $M_i(r^\prime , \phi ^\prime )$ are finite non-zero functions of $r^\prime$ and $\phi ^\prime$. However, as $R_2(\phi )=\tau _2 R(\phi )$ is a fictitious region, $\tau _2$ can achieve any arbitrary value. Therefore, by setting $\tau _2 \rightarrow \tau _3$ (i.e., $\delta \rightarrow 0$) as shown in Fig. 1c (evolved virtual space), the electric potential distribution inside the stretched region will be obtained by substituting Eq. (3) into the Laplace equation, $\left( \nabla \cdot \bar{\bar{\sigma }} \nabla \right) V=0$, as

4 $$\begin{aligned}&\frac{1}{\rho } M_1(r^\prime ,\phi ^\prime ) \frac{\partial V}{\partial \phi } + M_1(r^\prime ,\phi ^\prime ) \frac{\partial ^2 V}{\partial \rho ^2} + \frac{2\delta }{\rho }\sigma _{0}\frac{\tau _3(\tau _1-\tau _2)\left( \partial R(\phi )/\partial \phi \right) }{\delta r^\prime -\tau _3(\tau _1-\tau _2)R(\phi )} \frac{\partial ^2 V}{\partial \rho \partial \phi } + \frac{1}{\rho ^2} \delta ^2 M_2(r^\prime ,\phi ^\prime ) \frac{\partial ^2 V}{\partial \phi ^2}\nonumber \\&\frac{\delta }{\rho } \frac{\partial }{\partial \phi }\left[ \sigma _0\frac{\tau _3(\tau _1-\tau _2)\left( \partial R(\phi )/\partial \phi \right) }{\delta r^\prime -\tau _3(\tau _1-\tau _2)R(\phi )}\right] \frac{\partial V}{\partial \rho } =0 \Rightarrow \frac{1}{\rho } \frac{\partial V}{\partial \phi } + \frac{\partial ^2 V}{\partial \rho ^2}=0 \end{aligned}$$

It should be remarked that setting $\delta \rightarrow 0$, represents a transformation function that maps the region of $R_2(\phi )<r<R_3(\phi )$ with no volume (i.e., surface ) to the domain of $R_1(\phi )<r^\prime <R_3(\phi )$ that has a physical volume. This kind of volumeless transformation is known as null-transformation^27^. The obtained potential distribution of Eq. (4), is the same as that of a medium with the conductivity of $\sigma =$diag$[1/\Delta , \Delta , \Delta ]$, where $\Delta \rightarrow 0$ and diag[.] represents a diagonal matrix. This indicates that although $\sigma _{12}$ ($\sigma _{21}$) has non-zero values, their exact values are not important since eventually, the potential distribution is the same as that of a medium that has diagonal tensor of conductivity. Therefore, the final conductivities, which describe the performance of an arbitrary shape dc electric concentrator, will be achieved as

5 $$\begin{aligned} \bar{\bar{\sigma _s}}= \begin{bmatrix} 1/\Delta &{} 0\\ 0 &{} \Delta \\ \end{bmatrix} , \bar{\bar{\sigma _c}}= \begin{bmatrix} 1 &{} 0\\ 0 &{} 1 \end{bmatrix} =1 \end{aligned}$$

with $\Delta \rightarrow 0$. We have named the conductivity tensor of $\bar{\bar{\sigma _s}}$ as DNM which is an extreme anisotropic conductivity. Moreover, according to the expression of $\bar{\bar{\sigma _c}}$, the material of the inner section of concentrator is the same as that of the background medium. This will ease the fabrication process since one can utilize the same material for both regions of the background and inner section.

**Figure 2:**
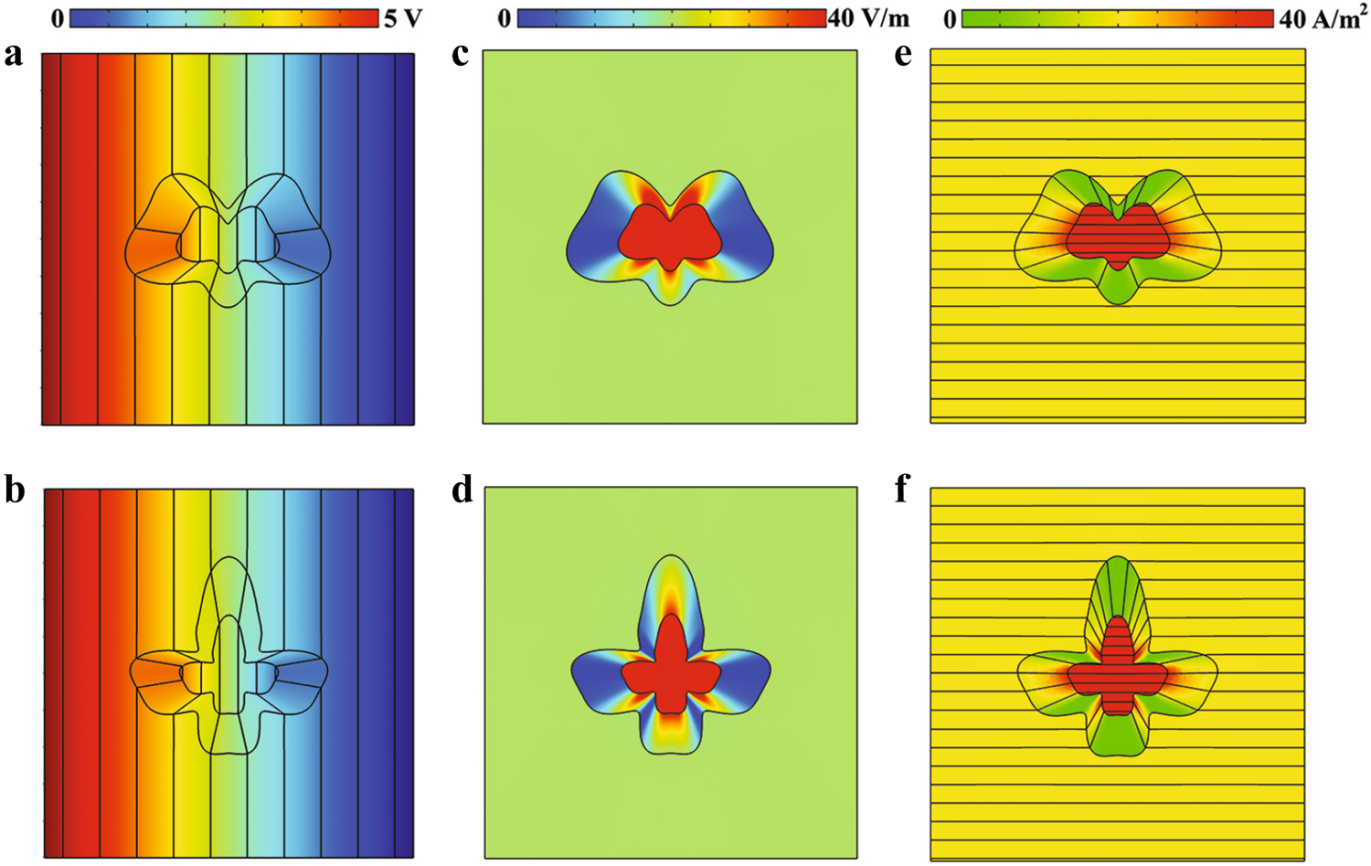
(**a**,**b**) The simulation result of electric-potential distributions of two arbitrary shape dc electric concentrators. (**c**,**d**) Their corresponding electric field distributions. (**e**,**f**) The steady current density distribution of the designed dc concentrators (black parallel thick lines indicate the steady current density).

### Numerical simulations

To outline the validity of the obtained conductivities of Eq. (5) and demonstrate its shape independent capability, we will study several arbitrary shape dc electric concentrators and examine their electric field, current density, and voltage distributions with COMSOL MULTIPHYSICS finite element solver. The solving area consists of a 0.5 m $\times$ 0.5 m square that its left side ($x=-0.25$ m), is connected to the potential of $V=5$ V and the right one ($x=+0.25$ m) is grounded, while its upper and lower sides ($y=\pm 0.25$ m) are set to be electric insulator ($n\cdot J=0$). Despite the fact that concentrating current density in an arbitrary region is of utmost importance, to the authors’ best knowledge, no systematic works have been yet proposed for this purpose. To this aim, while the contour coefficients of $\tau _1=0.5$, $\tau _2=0.99$ and $\tau _3=1$ are fixed, the cross section geometry of the concentrator (i.e., $R(\phi )$) is changed as it is shown in Fig. 2.

As can be seen from Fig. 2a,b, regardless of the geometry, electric potential distributions outside of the concentrator are not changed which indicates that the designed concentrators do not affect the background medium electric potential. Moreover, in the compressed region, the electric field is enhanced by the ratio of $\tau _2/\tau _1=1.98$ from $E_x=20$ V/m$^{-1}$ to 39.6 V/m$^{-1}$ as shown in Fig. 2c,d which well abides with the theoretical predictions. Furthermore, since the current density has a direct relation with the electric field as $\mathbf{J} =\bar{\bar{\sigma }}\cdot \mathbf{E}$, it would be also enhanced by the ratio of $\tau _2/\tau _1$ as it is shown in Fig. 2e,f from 20 A/m$^{-2}$ to 39.6 A/m$^{-2}$, while having no effects on the current distributions of the homogeneous space.

## Experimental verification

**Figure 3:**
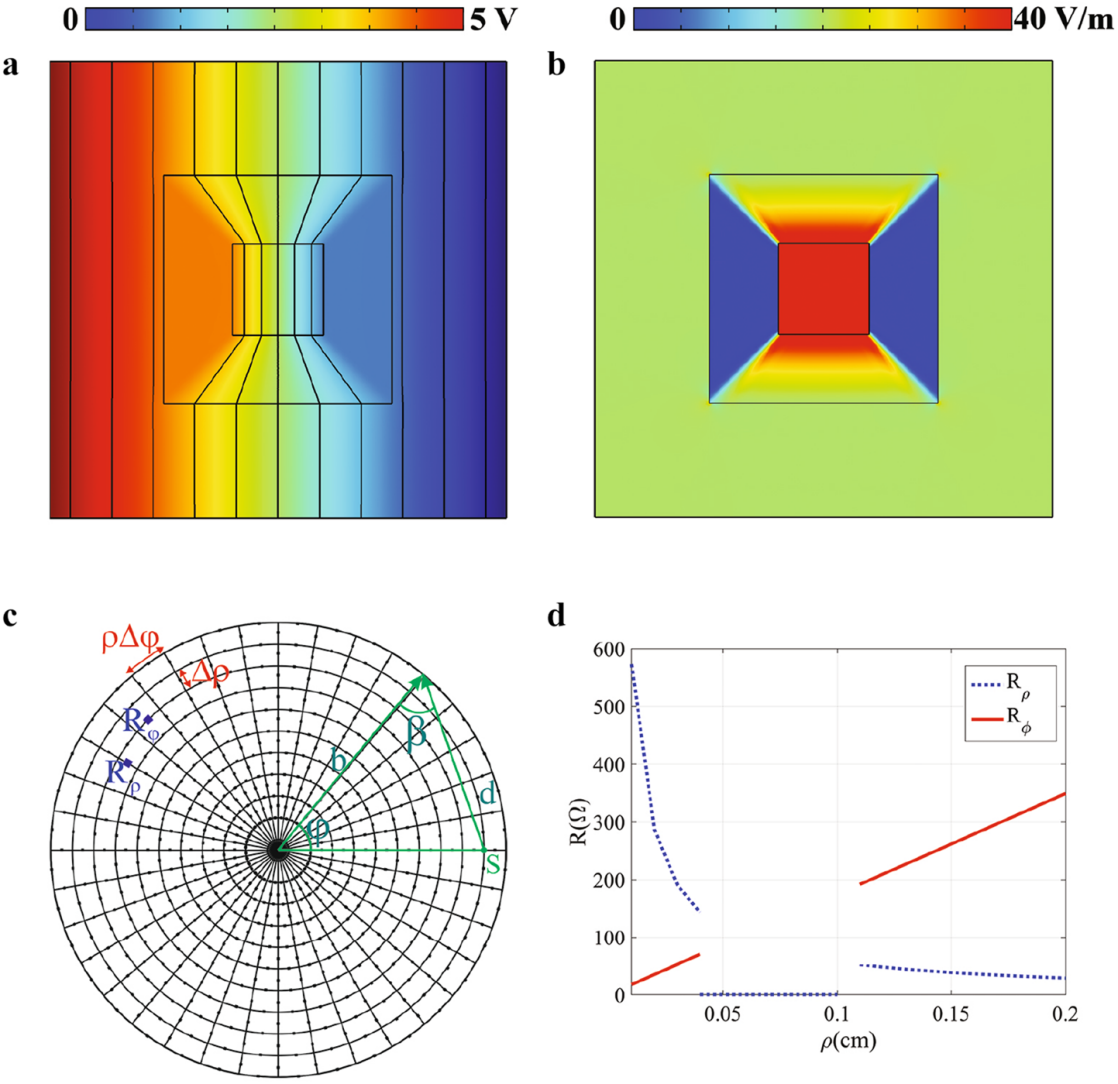
The simulation result of electric-potential distributions of a square shape dc electric concentrator. (**b**) The corresponding electric field distributions. (**c**) The schematic of the corresponding equivalent resistor network. (**d**) The obtained resistors for mimicking the desired conductivity behavior.

In order to authenticate the validity of the proposed material, we have designed and fabricated a square shape dc electric concentrator with the aid of analogy between electrical conductivity and resistor network. To this aim, it is assumed that the conductivity of the background medium is $\sigma _0=1$ S/m$^{-1}$ and the ratio of $\tau _2/\tau _1$ for the square shape concentrator is $\tau _2/\tau _1=2.72$. Thus, by utilizing the obtained conductivities of Eq. (5), the corresponding voltage and electric field of the designed concentrator will be achieved as shown in Fig. 3a,b. Although the required conductivities are difficult to be realized in nature, they could be emulated using the circuit theory if one discretizes the continuous material using the polar grids shown in Fig. 3c and uses two resistors with the values of $R_\rho =\frac{\Delta \rho }{\sigma _\rho \rho \Delta \phi h}$ and $R_\phi =\frac{\rho \Delta \phi }{\sigma _\phi \rho \Delta \rho h}$ for each of the elementary cell in the grid^12^. Thus, the anisotropic conductivity tensor can be implemented easily using different resistors in different directions as their obtained values are depicted in Fig. 3d. It is notable to mention that like the perfect matching layers in the time-varying problems, we have also designed matching resistors with the value of $R_\text {Matching}= \frac{d\times \left[ \ln (r_0)-\ln (r)\right] }{\sigma b h \cos (\beta ) \Delta \phi }$ in the outer ring to emulate an infinite material, which $r_0$ is the distance between the ground and the source point and the definitions of other geometrical parameters are provided in Fig. 3c. Moreover, the necessitating resistors for the stretching region will be achieved as $R_\rho =0$ and $R_\phi =\infty$ according to Eq. (4), which could be realized using the short and open circuits in the resistor network, respectively.

**Figure 4:**
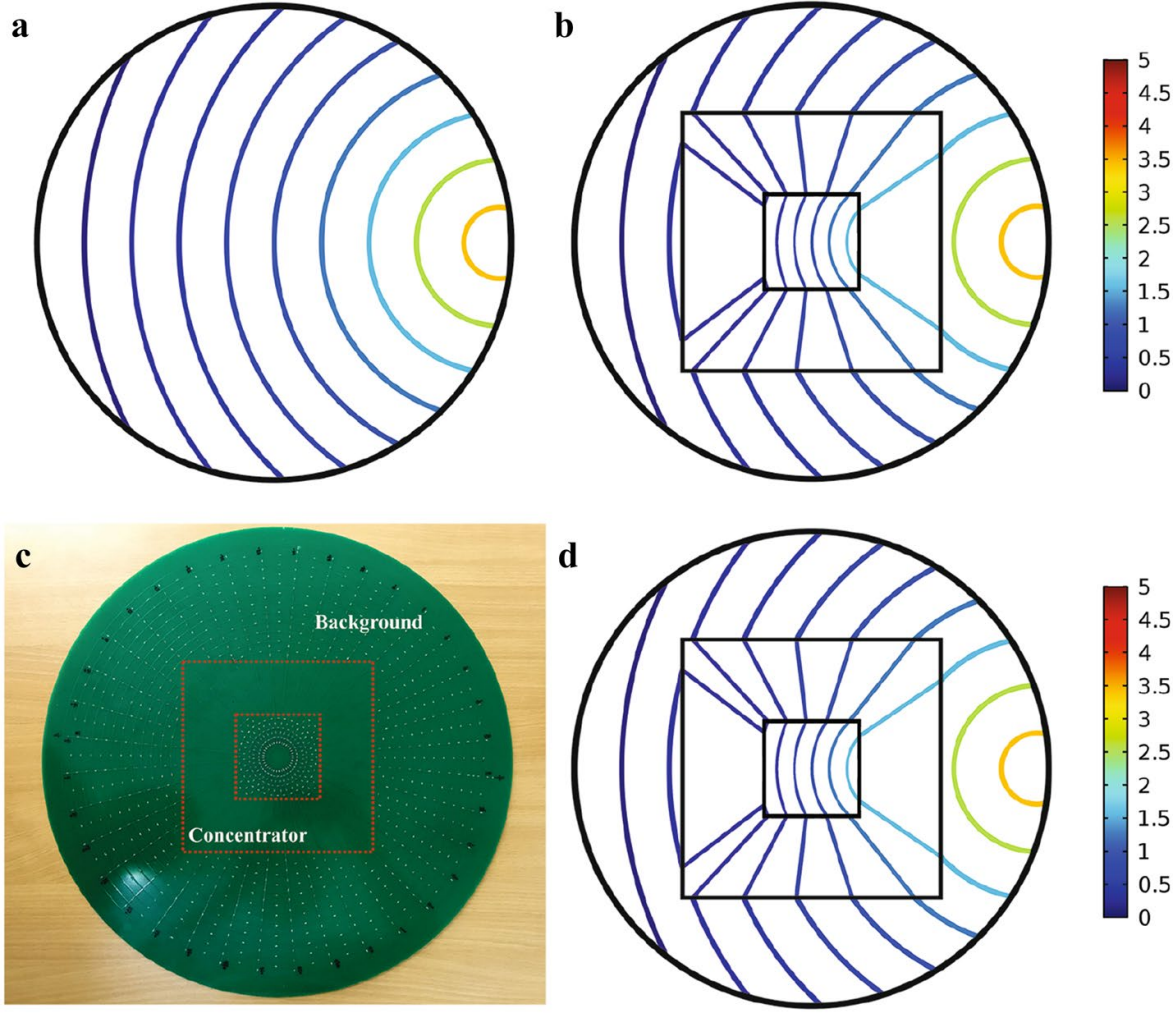
The ADS simulation results of equipotential lines. (**a**) Homogeneous medium when no dc electric concentrator is exploited and (**b**) when the device is utilized. (**c**) The photograph of the fabricated square shape dc electric concentrator. (**d**) The experimental results of equipotential lines when the square shape dc electric concentrator is utilized.

To demonstrate the effectiveness of our design, we firstly simulate dc electric concentrator in a homogeneous background material based on the resistor network of Fig. 3d using the commercial software of Agilent Advanced Design System (ADS). That is, the functionality of the realized concentrator will be verified by ADS via obtaining the voltage of resistor network nodes and then calculating their corresponding currents by Ohm’s law. Following the above-mentioned design procedure, the background material is discretized into $20 \times 36$ cells using the polar grid and its voltage distribution is depicted in Fig. 4a.

As it was expected, the equipotential lines when no electric concentrator is exploited are concentric circles with the center of the source location, which in the simulation environment it is located at the 19-th layer. However, when the square shape concentrator is utilized, the equipotential lines will be transferred point-to-point from the outer interface to the inner one in the stretching region while the ones outside the concentrator keep the original equipotential lines as those in the homogeneous material as shown in Fig. 4b. The fabricated sample of the arbitrary shape dc electric concentrator is also shown in Fig. 4c. Since in experiment, the potential is much easier to measure than the current density, we have measured potential distributions and its results are given in Fig 4d. It is evident that the fabricated sample demonstrates an excellent concentrating performance compared with the numerical simulation results which corroborate the validity of the presented design principle. It should be remarked that the slight discrepancies in the numerical results shown in Fig. 4b with the measurement results given in Fig. 4d is due to the errors of practical resistors since the utilized resistors are not exactly the same as the ones obtained from theoretical investigations.

## Discussion

In this work, based on the TE methodology and with the aid of null transformation, we have proposed a feasible method to achieve an arbitrary shape dc electric field concentrator via a simple homogeneous conductivity, named as DNM. The proposed concentrator can enhance the static electric fields in a given region while the external fields keep undisturbed. In contrary to the previous studies, the obtained material is independent of the input geometry. That is, if the concentrator shape is changed, the same DNM could be exploited and there is no demand to recalculate the new material. In addition, based on the analogy between conductivity and resistor network, the obtained conductivities were then realized with low-cost and commercial resistors. To authenticate the idea, we have designed and fabricated a square shape dc electric concentrator based on the realized resistors. It was observed that the measurement results exhibit good agreement with the numerical simulations and theoretical predictions. The proposed design principle could be utilized to fabricate concentrators that are applicable in the electric impedance tomography and e-skin devices.

## Methods

In particular, we have introduced a new kind of transformation function, null-transformation, which leads to an extreme anisotropic conductivity named as DNM, which has been shown that is independent of the concentrator geometry. This has been verified by simulating different shape concentrators and examined their electric field and voltage distributions with COMSOL MULTIPHYSICS solver. That is, after calculating and assigning the demanding materials to their corresponding regions, the voltage distribution (with its equipotential lines) and the electric field distribution have been obtained and analyzed with COMSOL MULTIPHYSICS solver. Afterwards, a square shape dc electric concentrator has been realized with the aid of low-cost resistor network. To examine the performance of the implemented dc electric concentrator, we have used Advanced Design System (ADS) solver. In particular, in the ADS solver, we have established a resistor network that its topology is shown in Fig. 3c and each of its branches consist of resistors with the values given in Fig. 3d. A dc supply voltage with the magnitude of 5 V is then applied to the 19-th layer of such a network and the voltage of each node as well as their currents have been evaluated in the ADS to form Fig. 4b. In other words, the functionality of the realized concentrator has been numerically verified by ADS via obtaining the nodes voltages of the resistor network and then calculating their corresponding currents by Ohm’s law. In addition to the numerical simulations, we have fabricated the resistor network on a printed circuit board (PCB) that has a thickness of 1.6 mm, while contains 20 concentric layers and 36 nodes in the radial and tangential directions, respectively. According to the given geometry, we have dedicated an area that covers 4 layers for the compressed region, while another 7 layers have been occupied by the stretched domain. The remaining 9 layers are utilized for mimicking the behavior of the background material. It should be noted that the exploited resistors for the fabricated sample are commercial SMD resistors with an accuracy of 1$\%$. Furthermore, a dc source with the magnitude of 5 V has connected to the 19-th layer of the network to supply the voltage of the circuit while measuring the voltage at each node has been done using a 4.5-digit multimeter.
